# Postoperative anaemia might be a risk factor for postoperative delirium and prolonged hospital stay: A secondary analysis of a prospective cohort study

**DOI:** 10.1371/journal.pone.0229325

**Published:** 2020-02-21

**Authors:** Julius Valentin Kunz, Claudia D. Spies, Anna Bichmann, Miriam Sieg, Anika Mueller

**Affiliations:** 1 Department of Anesthesiology and Operative Intensive Care Medicine, Charité Campus Mitte and Campus Virchow Klinikum, Charité–Universitaetsmedizin Berlin, Corporate Member of Freie Universität Berlin, Humboldt-Universität zu Berlin, and Berlin Institute of Health, Berlin, Germany; 2 Department of Nephrology and Medical Intensive Care, Charité–Universitaetsmedizin Berlin, Corporate Member of Freie Universität Berlin, Humboldt-Universität zu Berlin, and Berlin Institute of Health, Berlin, Germany; 3 Institute of Biometry and Clinical Epidemiology, Charité–Universitaetsmedizin Berlin Corporate Member of Freie Universität Berlin, Humboldt-Universität zu Berlin, and Berlin Institute of Health, Berlin, Germany; 4 Berlin Institute of Health (BIH), Berlin, Germany; Heidelberg University Hospital, GERMANY

## Abstract

**Background:**

Postoperative anaemia is a frequent surgical complication and in contrast to preoperative anaemia has not been validated in relation to mortality, morbidity and its associated health economic effect. Postoperative anaemia can predispose postoperative delirium through impairment of cerebral oxygenation. The aim of this secondary analysis is to investigate the association of postoperative anaemia in accordance with the sex specific World Health Organization definition of anaemia to postoperative delirium and its impact on the duration of hospital stay.

**Methods:**

A secondary analysis of the prospective multicentric observational CESARO-study was conducted. 800 adult patients undergoing elective surgery were enrolled from various operative disciplines across seven hospitals ranging from university hospitals, district general hospitals to specialist clinics of minimally invasive surgery in Germany. Patients were classified as anaemic according to the World Health Organization parameters, setting the haemoglobin level cut off below 12g/dl for females and below 13g/dl for males. Focus of the investigation were patients with acute anaemia. Patients with present preoperative anaemia or missing haemoglobin measurement were excluded from the sample set. Delirium screening was established postoperatively for at least 24 hours and up to three days, applying the validated Nursing Delirium Screening Scale.

**Results:**

The initial sample set contained 800 patients of which 183 were suitable for analysis in the study. Ninety out of 183 (49.2%) suffered from postoperative anaemia. Ten out of 93 (10.9%) patients without postoperative anaemia developed a postoperative delirium. In the group with postoperative anaemia, 28 (38.4%) out of 90 patients suffered from postoperative delirium (odds ratio 3.949, 95% confidence interval, (1.358–11.480)) after adjustment for NYHA-stadium, severity of surgery, cutting/suture time, duration of anaesthesia, transfusion of packed red cells and sedation status with Richmond Agitation Scale after surgery.

Additionally, patients who suffered from postoperative anaemia showed a significantly longer duration of hospitalisation (7.75 vs. 12.42 days, odds ratio = 1.186, 95% confidence interval, 1.083–1.299, after adjustments).

**Conclusion:**

The study results reveal that postoperative anaemia is not only a frequent postsurgical complication with an incidence probability of almost 50%, but could also be associated with a postoperative delirium and a prolonged hospitalisation.

## Introduction

According to the definition of the World Health Organization (WHO) anaemia exists when the haemoglobin (HB) level is below 12 g/dl for females and below 13 g/dl for males [1].

A meta-analysis of almost one million patients undergoing major non-cardiac (vascular, orthopaedic, spinal and upper gastrointestinal) and cardiac surgery worldwide showed a preoperative anaemia prevalence of 39.2% and an association with an increased rate of postoperative complications, as well as a higher 30-day morbidity and mortality rate [2]. The postoperative complications associated with preoperative anaemia range from stroke, infection [2], pneumonia, sepsis, venous thromboembolism, wound healing disturbance and acute myocardial infarction to cardiac arrest [3]. In regards to postoperative anaemia, a multicentre observational study stated a prevalence of 85.8% among 1534 patients undergoing major elective orthopaedic surgery, highlighting a high-risk group [4]. Further complications, including acute kidney injury (AKI), have been demonstrated for postoperative anaemic patients, presumably caused by a lack of oxygen supply due to reduced oxygen carrying capacity [5]. AKI directly correlates with a higher mortality rate and higher hospital expenses along with other causes leading to a prolonged length of stay in the hospital [6]. A reduced oxygen carrying capacity could also affect the brain, leading possibly to a delirium [7]. The Diagnostic and Statistical Manual of Mental Disorder 5 (DSM-5) criteria defines delirium as an acute and fluctuating disturbance in attention and cognition, which is not based on a pre-existing neurocognitive disorder[8]. A delirium often occurs postoperatively with an incidence of up to 74% [7].

Postoperative delirium (POD) is associated with prolonged hospitalisation [9], impairments of daily life [10] and onset of posttraumatic stress disorder [11]. Other implications are long-term cognitive decline [12] and dementia [13] which can develop after a POD. Furthermore, POD itself is an independent risk factor for postoperative mortality [14, 15]. Postulates are that the aetiology of POD is multifactorial with”a complex interrelationship between predisposing and precipitating factors”[16].

Considering that preventing POD is by far more effective than its treatment [17], it is particularly important to identify predisposing- and precipitating factors in order to avoid it. To achieve this, recommendations on POD risk factors need to be published. One of the considered risk factors for POD is intraoperative bleeding as stated by the European Society of Anaesthesiology in a recently published evidence- and consensus-based guideline of POD. However, no HB threshold has been mentioned [18].

The aim of this secondary analysis is to investigate the association of postoperative anaemia in accordance with the sex specific WHO thresholds for POD and duration of hospital stay.

## Methods

### Study design and setting

Data collection has been performed in the multicentre observational study CESARO which has recently been published [19]. The study was initiated by the Department of Anaesthesiology and Operative intensive Care Medicine at the Charité–Universitätsmedizin Berlin. Participant centres consisted of the university hospitals of Berlin, Bochum, Heidelberg, Munich, Regensburg, Ulm and Würzburg. The district general hospital of Wetzlar and the specialist clinic of minimally invasive surgery Berlin took part in the study as well. All centres are located in Germany (see Clinicaltrials.gov ID: NCT01964274).

The observational study was approved by the independent Charité Ethics Committee, Charité–Universitätsmedizin Berlin, Germany and was registered under ref.: EA1/220/13 on 14th August 2013. All patients had a pre-operation discussion, gave their written consent and local data privacy regulations were complied with.

Primary endpoint in the original observational study, both acetyl- and butyrylcholinesterase activity (AChE and BuChE), were investigated in the patient’s blood samples, with and without POD in the early period after surgery [19]. The data were collected between October 2013 and August 2016 by the anaesthesiology departments of the participant centres. Perioperative HB measurement was a secondary endpoint.

### Participants

A logistic regression analysis was conducted for the sample group of the original study which required 797 patients. The group consisted of patients 18 years of age or older, undergoing any elective surgery in a broad range of operative disciplines with admission in a post-anaesthesia care unit (PACU) and an inpatient care for at least twenty-four hours. Criteria for exclusion from the sample group were AChE or BuChE deficiencies, illiterate patient, inability to speak English or German, impairment of vision or hearing and current participation in other clinical trials.

### Data assessment

The data collected included sex, age, medical history, smoking status, body mass index (BMI), heart failure according to the NYHA classification system and medication with anticholinergic drugs.

The surgery and anaesthetically related characteristics assessed in the original study are: preoperative physical status (ASA Classification System), duration of anaesthesia, severity and type of surgery, the amount of infusion and blood product transfusion during surgery. The severity of surgery was categorized in accordance with the POSSUM-Score (*P*hysiological and *O*perative *S*everity Score for the enumeration of *M*ortality and morbidity) [20].

The HB level before and after surgery as well as the length of admission were obtained from medical records. The project group also recorded the patient´s Glasgow Coma Scale (GCS)[21], the sedation status with the Richmond Agitation Scale (RASS)[22], incidences of postoperative nausea and vomiting (PONV) and vital parameters while admission, ten minutes after and at discharge in/off post-anaesthesia care unit (PACU). Hypotension was defined as middle arterial pressure (MAP) below 60 mmHg.

All data were recorded by means of an online-based case report form.

### Diagnosis of delirium

Delirium was assessed by using the Nursing Delirium Screening Scale (NU-DESC). Members of a research team supervised by delirium specialists, consisting of physicians, medical students and study nurses, conducted the assessment.

NU-DESC is a five-item scale containing orientation, behaviour, communication, illusion and psychomotor retardation. Every component is rated on a scale from zero to two points. A cumulated score of two or more points is considered as POD positive [23].

The DSM-5 criteria are considered to be the gold standard for delirium diagnosis, however, it is protracted, requires extensive training and is often not practicable in a busy recovery room.

Because of the multicentric design of the original study with its high sample size, multiple delirium screening per patient and large team of assistance in data collection the NU-DESC method was used instead. NU-DESC was evaluated in the PACU showing a sensitivity of 95% and specificity of 87% with an average completion time of one minute.[24]

In postoperative inpatient treatment, with multiple screenings per day, NU-DESC had a sensitivity of 98% and specificity of 92%.[25]

Delirium was screened by the research team on admission in the PACU, on discharge from the PACU and on postoperative day one and three.

### Statistical analysis

All patients with recorded pre- and post-operative HB level were eligible for statistical analyses.

Patients who suffered from preoperative anaemia were excluded.

By using either a Chi-Square-Test, Mann-Whitney-U Test (when data is not normal distributed) or T-Test (normal distribution), the groups of patients with and without postoperative anaemia were analysed. A p-value of less than or equal to 0.05 was regarded as statistically significant. Due to the exploratory character of the analysis, no adjustment for multiple testing has been performed.

Results were depicted as mean ± standard deviation (SD) and frequencies with percentages (%). Ordinal variables were expressed as median with interquartile range [IQR].

Multiple logistic regression was carried out with statistically significant differences, to examine the associations of postoperative anaemia. Variables were selected if univariate tests showed statistical significance.

SPSS Version 21 was used for all statistical analysis and all indicated p-values were two-sided.

## Results

815 patients were considered as eligible for the study. However, 632 patients were excluded due to incomplete HB measurement (481) and preoperative anaemia (136) as well as 15 participants who left the study. Out of the remaining 183 participants 90 suffered from postoperative anaemia (according to WHO definition).

**Fig 1:**
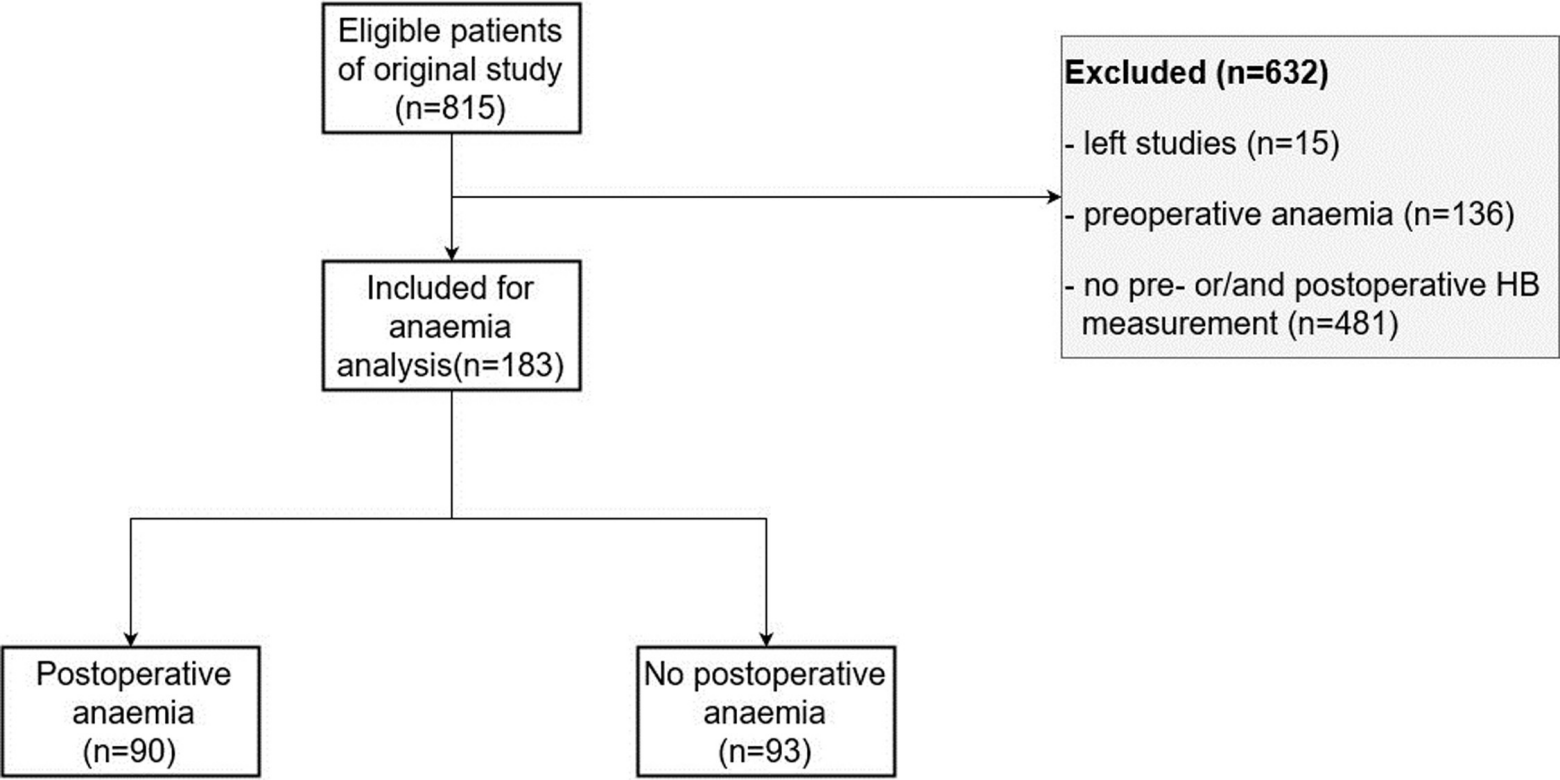
Flowchart.

The final data set included 183 patients (Fig 1). The baseline characteristics of all included patients are illustrated in Table 1.

**Table 1:** Characteristics of all included patients.

	Postoperative anaemia n = 90		No postoperative anaemia n = 93			p-value
Demographics						
Age [years]	68.5	±14.9	66.1	±13		0.260
Female Gender	39	43.3%	47	50.5%		0.329
BMI [kg/m^2^]	27.5	±5.9	27.5	±6.9		0.995
Smoker	15	16.7%	23	24.7%		0.179
Daily consume of alcohol	7	8.3%	14	18.2%		0.064
Atrial fibrillation	18	20.0%	10	10.8%		0.082
ASA	3	[1]	3	[1]		0.097
NYHA	0	[2]	0	[0]	<	0.001*
Intraoperative parameters						
Surgical Risk	3	[2]	2	[1]		0.001*
Hypotension before anaesthesia	0	0%	0	0%		-
Heart rate before anaesthesia [bpm]	70	[18]	74	[14]		0.85
Crystalloid infusion in [ml]	2035.3	±1317.0	1984	±1566		0.820
Colloid infusion [ml]	198.1	±512.7	178	±444		0.658
Transfusion of packed red cells	14	16.5%	6	6.7%		0.042*
Transfusion of fresh-frozen-plasma	10	11.8%	6	6.7%		0.242
Transfusion of thrombocyte concentrate	7	8.2%	3	3.2%		0.163
Duration of anaesthesia [min]	217.9	±92.8	165.3	±82.7	<	0.001*
Duration of surgery [min]	140.1	±80.6	101.2	±68.9		0.001*
Postoperative parameters						
POD	28	38.4%	10	10.9%	<	0.001*
PONV	17	18.9%	14	15.1%		0.489
Hypotension on admission in RR/PACU	4	4.4%	0	(0%)		0.58
Heart rate on admission in RR/PACU [bpm]	85	[20]	80	[20]		0.14
RASS on admission in RR/PACU	-1	[3]	0	[1]	<	0.001*
GCS on admission in RR/PACU	15	[1]	15	[0]		0.001*
Duration of hospital admission [days]	12.4	±8.7	7.8	±4.4	<	0.001*

Study Group; No of Patients (%), median [IQR] or mean (± SD); IQR = interquartile range; BMI = body mass index; ml = millilitre; surgical risk measured by POSSUM; min = minutes; POD = postoperative delirium; PONV = postoperative nausea and vomiting; bpm = beats per minute; RR = recovery room; PACU = post-anaesthesia care unit; RASS = Richmond Agitation Scale; GCS = Glasgow Coma Scale; mv = missing values;

*p < 0.05.

Table 1 demonstrates that patients with postoperative anaemia have a higher NYHA Score, a significant longer anaesthesia and cutting/suture time, a more severe procedure (POSSUM Score), a lower grade of vigilance (GCS) and a deeper sedation status (RASS) on admission in PACU. Furthermore, patients with postoperative anaemia had a transfusion of packed red cells (PRC) more often.

Additionally, POD occurred at a higher rate of 38.4% for inpatients who suffered from postoperative anaemia in comparison to 10.9% for inpatients without postoperative anaemia as well as a prolonged length of hospitalisation of 12.42 days compared to 7.75 days.

Ninety events of postoperative anaemia were recorded and nine categories were statistically significant. To keep the risk of overfitting low, we were able to include nine independent variables in the multiple logistic regression according to ten events per variable rule [26]. Postoperative GCS was excluded due to numerous missing values.

In the multiple logistic regression, the groups did not differ statistically significant in NYHA Score, surgical risk, transfusion of PRC, duration of anaesthesia, cutting/suture time and RASS on admission in PACU. Whereas the higher incidence of postoperative delirium (p = 0.012) and the duration of hospitalisation (p = <0.001) were statistically significant (**Table 2)**. The chance to develop a POD is 3.949 (95% CI, (1.358–11.480), after adjustments (AAs)) times higher for inpatients with postoperative anaemia.

**Table 2:** Results from multiple logistic regression, associations of postoperative anaemia.

Covariables		p-values	Odds Ratio (95%- confidence interval)
Duration of hospitalisation POD	<	0.001* 0.012*	1.186 (1.083–1.1.299) 3.949 (1.358–11.480)
NYHA Surgical risk Transfusion of packed red cells		0.651 0.610 0.643	1.120 (0.685–1.831) 1.141 (0.688–1.892) 0.708 (0.165–3.046)
Duration of anesthesia Duration of surgery RASS on admission in RR/PACU		0.260 0.147 0.133	0.990 (0.973–1.007) 1.015 (0.995–1.035) 0.764 (0.538–1.085)

surgical risk measured by POSSUM; POD = postoperative delirium; RR = recovery room; PACU = post-anaesthesia care unit; RASS = Richmond Agitation Scale;

*p < 0.05.

The duration of hospitalisation was 7.75 days for inpatients without anaemia vs. 12.42 days for anaemic inpatients (OR = 1.186, 95% CI, 1.083–1.299, AAs).

## Discussion

The secondary analysis revealed that 90 of 183 patients suffered from postoperative anaemia. Patients who suffer from postoperative anaemia have an increased likelihood to sustain POD by almost four times AAs for their stadium of heart failure, severity of surgery, postoperative blood pressure, transfusion of PRC, duration of anaesthesia, heart rate before anaesthesia and RASS on admission in PACU. Despite the supervision by delirium specialists during data assessment, POD, if occurred, was not confirmed according to DSM-5 criteria. Hence, it is to be considered that this might have led to false positive testing in some cases. NU-DESC false positive POD testing was at 12,8% DESC and DSM criteria was recommended for confirmation [24].

The connecting link between postoperative anaemia and onset of POD could be an inadequate cerebral oxidative metabolism which can cause, among others, imbalance of neurotransmitter, collapse of the brain barrier and neuroinflammation which are under discussion to be a trigger for delirium [7]. In a study of 101 intensive care unit patients where oxygenation was measured by HB, haematocrit and pulse oximetry came to the conclusion that the occurrence of delirium during intensive care admission was associated with poorer oxygenation before onset [27].

Regarding the above, no surrogate parameter was used in the study cohort to clarify if anaemia caused a decreased cerebral oxygenation.

Due to the heterogenous definition of anaemia it proofs to be challenging to compare earlier studies with the current one, which investigated postoperative anaemia and POD. An association between postoperative anaemia with a HB level equal to and below 9.7 g/dl [28] or postoperative haematocrit ≤ 30% [29], [30, 31] and POD has already been established. According to another study there is no significant association between POD and variable severity of anaemia comparing 8–10 g/dl and above 10 g/dl [32].

To address the heterogeneity of definition of anaemia in prior studies, the worldwide validated sex specific WHO definition of anaemia was used and successfully demonstrated its association with POD.

POD triggered by anaemia might have more extensive clinical consequences than POD of other origin, however, this extends beyond the scope of this research but should be investigated in further studies.

A recent study examined clinical phenotypes of delirium during critical illness of 1040 patients and their severity of subsequent long-term cognitive impairment in a prospective cohort design. The study distinguished between sedative-associated, hypoxic, septic, metabolic and unclassified delirium. One of the findings was that the duration of hypoxic, septic and sedative-associated delirium is associated with long-term cognitive impairment. Whereas the duration of a metabolic delirium is not. Hypoxic delirium was defined as hypoxemia or shock [33]. However, it is questionable if an anaemia related delirium has the same long-term impacts.

Another key finding of the analysis is the significantly prolonged hospital stay of patients diagnosed with postoperative anaemia (7.75 compared to 12.42 days) AAs. A prospective observation of 550 patients who underwent surgery due to a hip fracture supports this finding. Whereas a higher postoperative HB level shortens the length of hospital admission [34].

In the analysis it turned out that one of the avoidable causes for postoperative anaemia is iron deficiency. According to a prospective study on patients undergoing hip surgery [35], iron deficiency without anaemia leads to a more frequent preoperative transfusion of blood cells [36], a lowered intravenous iron supplementation transfusion rate, 30-day mortality rate and shortened duration of hospitalisation. Additionally, the majority of surgical patients who had iron deficiency without anaemia became anaemic after surgery [4]. This poses the question whether a potentially easy treatable iron deficiency is co-responsible for postoperative anaemia among the study cohort. Unfortunately, no data were collected on iron status for further evaluation.

Blood transfusion, regarded as the quickest and most effective treatment of acute anaemia, is discussed highly controversial in terms of POD. There are indications for both, increasing [32, 37] and decreasing [28, 38] POD incidences.

According to this analysis, perioperative transfusion of erythrocyte concentrates AAs, displayed in Table 2, had no statistically significant influence on postoperative anaemia.

Also, considering the risks of blood transfusion (e.g. higher rate of mortality and morbidity[39], neuroinflammation and cognitive impairment [40]), the utilization of effective remedies like iron infusions [35, 41], tranexamic acid[42], bipolar sealer [43] and cell-saver [44] to avoid allogenic blood transfusion should be fully exhausted [45]. For example, a perioperative iron therapy showed a lower 30-day mortality rate and a reduction in blood transfusions [46]. The perioperative use of tranexamic acid preserved a higher postoperative HB level reducing the length of hospitalisation [47].

Postoperative anaemia is likely to have even more extensive consequence. A study of 152,757 inpatient treatments highlighted that patients who were discharged with a mild anaemia (HB level of 11-12g/dl) showed a higher risk of re-admission within 30 days after discharge [48]. It would have been of great interest to extend the data set of this study to investigate the HB level at discharge and related re-admission rates.

## Limitations of this secondary analysis

Perioperative HB measurement was a secondary endpoint in the study, leading to a high rate of missing values in the dataset and the applied methods do not necessarily establish causality.

Delirium was assessed using NU-DESC, which is not considered as the gold standard, however proved to be effective for data collection due to its lower application level. Even though, it showed a very sensitive score in the PACU with a high specificity and a 12,8% false positive delirium rate. For this purpose, confirmation with DSM-5 criteria was recommended but not performed in the course of this study[24].

Blood transfusion itself can trigger neuroinflammation and predispose patients to develop POD [40]. Hence, statistical analysis could potentially be skewed.

In the evidence- and consensus-based guidelines of POD, published in 2017, screening for POD was recommended until the fifth postoperative day [18]. Due to the fact that data have been collected between October 2013 and August 2016, POD was screened just until the third postoperative day.

## Conclusion

Postoperative anaemia occurs frequently for surgical patients. According to the findings of this analysis, patients with postoperative anaemia potentially have a significantly higher risk of POD and a prolonged hospital stay. However, whether the prevention of postoperative anaemia would lead to a lower incidence of POD and shorten hospitalisation remains unclear and could be subject to further studies. Additional trials are needed to standardize an approach for the detection, prevention and treatment of anaemia as a possible risk factor for POD and prolonged hospital stay.

## Supporting information

S1 File Overview of the Study Protocol in German. (PDF)

S2 File Overview of the Study Protocol in English. (PDF)
