# Supplementary material for: Postoperative anaemia might be a risk factor for postoperative delirium and prolonged hospital stay: A secondary analysis of a prospective cohort study
Source: PLoS One. 2020 Feb 21;15(2):e0229325. doi: 10.1371/journal.pone.0229325 (PMC7034819; doi:10.1371/journal.pone.0229325)
Supplement: S1 File — (PDF) [file pone.0229325.s001.pdf]

Studienprotokoll

Visiten	Screening/ Baseline	Narkose-einleitung	Operation	Aufnahme PACU	Entlassung PACU	Nach 24h	Nach 2-3 d
**Tage** / **Unter-suchungen**	**-2 / -1 Tage**	**OP-Tag**	**OP-Tag**	**OP-Tag**	**OP-Tag / 1. POD**	**1. POD**	**2. - 3. POD**
Überprüfung der Ein- und Ausschlusskriterien	✓						
Anamnese/Diagnosen inkl. früherer Therapien	✓						
Einverständniserklärung zur Studie	✓						
Körperliche Untersuchung	✓						
Demografische Daten	✓						
Vitalparameter	✓	✓	✓	✓	✓	✓	✓
Begleitmedikation	✓	✓	✓	✓	✓	✓	✓
Behandlungsdaten			✓	✓	✓	✓	✓
Begleiterkrankungen/ Organkomplikationen	✓	✓	✓	✓	✓	✓	✓
Cholinesterase-Aktivität	✓	✓		✓	✓	✓	✓
Delirscreening (Nu-DESC)	✓	✓		✓	✓	✓	✓
Schmerzen (NRS)	✓	✓		✓	✓	✓	✓

POD = postoperative day, PACU = post-anaesthesia care unit, NU-DESC = Nursing Delirium Screening Scale, NRS = numeric rating-scale
