# Supplementary material for: Postoperative anaemia might be a risk factor for postoperative delirium and prolonged hospital stay: A secondary analysis of a prospective cohort study
Source: PLoS One. 2020 Feb 21;15(2):e0229325. doi: 10.1371/journal.pone.0229325 (PMC7034819; doi:10.1371/journal.pone.0229325)
Supplement: S2 File — (PDF) [file pone.0229325.s002.pdf]

Study protocol

Assessment	Screening/ baseline	Narcosis induction	Surgery	Admission in PACU	Discharge of PACU	24h after	2-3 days after
Date / Assessment	**-2 / -1 days to surgery**	**day of surgery**	**day of surgery**	**day of surgery**	**day of surgery / 1. POD**	**1. POD**	**2. - 3. POD**
Ex- and inlusion criteria	✓						
Medical history including previous medical therapies	✓						
Pre-operation discussion and written consent	✓						
Physical examination	✓						
Demographics	✓						
Vital parameters	✓	✓	✓	✓	✓	✓	✓
Medication	✓	✓	✓	✓	✓	✓	✓
Perioperative parameters			✓	✓	✓	✓	✓
Comorbidities/ organcomplications	✓	✓	✓	✓	✓	✓	✓
Cholinesterase activity	✓	✓		✓	✓	✓	✓
Delirium assessment NU-DESC	✓	✓		✓	✓	✓	✓
Pain (NRS)	✓	✓		✓	✓	✓	✓

POD = postoperative day, PACU = post-anaesthesia care unit, NU-DESC = Nursing Delirium Screening Scale, NRS = numeric rating-scale
